# Evaluation of PAMAM Dendrimer-Stabilized Gold Nanoparticles: Two-Stage Procedure Synthesis and Toxicity Assessment in MCF-7 Breast Cancer Cells

**DOI:** 10.3390/molecules30092024

**Published:** 2025-05-02

**Authors:** Agnieszka Maria Kołodziejczyk, Magdalena Grala, Łukasz Kołodziejczyk

**Affiliations:** 1 Nanomaterial Structural Research Laboratory and Molecular and Nanostructural Biophysics Laboratory, Bionanopark Ltd., Dubois 114/116, 93-465 Lodz, Poland; magdalena.grala@p.lodz.pl; 2 Food Science Department, Faculty of Pharmacy, Medical University of Lodz, Muszynskiego 1, 90-151 Lodz, Poland; 3 Department of Chemical and Molecular Engineering, Faculty of Process and Environmental Engineering, Lodz University of Technology, Wolczanska 213, 93-005 Lodz, Poland; 4 Institute of Materials Science and Engineering, Lodz University of Technology, Stefanowskiego 1/15, 90-537 Lodz, Poland

**Keywords:** gold nanoparticles synthesis, AuNPs, PAMAM G4 dendrimers, atomic force microscopy, XTT tetrazolium salt reduction tests, cellular viability

## Abstract

Gold nanoparticles stabilized with polyamidoamine dendrimers are one of the potential candidates for use as a contrast agent in computed tomography and a drug delivery agent. This work demonstrates a rapid, two-step synthesis of such complexes, which are size-stable for up to 18 months. The first step of the synthesis involves a short sonication of gold (III) chloride hydrate with polyamidoamine dendrimers of the fourth generation, while the second step uses microwaves to reduce gold (III) chloride hydrate with sodium citrate. The developed synthesis method enables rapid production of spherical and monodisperse gold nanoparticles stabilized with polyamidoamine dendrimers. Physicochemical characterization of the gold nanoparticle-polyamidoamine dendrimers complexes was performed using ultraviolet-visible spectroscopy, dynamic light scattering technique, infrared spectroscopy, atomic force microscopy, and transmission electron microscopy. The toxicity of synthesized complexes on the breast cancer MCF-7 cell line has been studied using the tetrazolium salt reduction test. The produced gold nanoparticles revealed lower toxicity levels on the MCF-7 cell line after 18 months from synthesis compared with newly synthesized colloids. Synthesized gold nanoparticles stabilized with dendrimers and commercially available gold nanoparticles stabilized with sodium citrate show similar toxicity levels on breast cancer cells.

## 1. Introduction

The field of cancer theranostics has seen the advent of nanoparticles, particularly gold nanoparticles (AuNPs), as a promising avenue for targeted and controlled drug delivery. The effective application of these nanoparticles, as well as other nanostructures such as carbon nanotubes, dendrimers, liposomes, and micelles, hinges on their physicochemical properties such as size, shape, and surface characteristics [1,2]. Among the different types of nanoparticles, gold nanoparticles have been demonstrated to inhibit proliferation and induce cell death in different types of cancer cell lines [3,4,5,6,7,8].

Gold nanoparticles present numerous benefits, including prolonged circulation time, straightforward modification with ligands that can be identified by cancer cell surface receptors [9], and improved uptake via receptor-mediated endocytosis. These features starkly contrast with traditional cancer treatments like surgery, chemotherapy, and radiation therapy, which often do not meet expectations. The mentioned therapies are often less effective against advanced or metastatic cancers, where the disease has spread to other parts of the body. These limitations highlight the need for more targeted and innovative approaches, such as those involving nanoparticles and other advanced technologies. AuNPs are utilized in targeted drug delivery therapies [10], significantly boosting therapeutic effects while reducing side effects when used in controlled drug delivery systems. Additionally, they serve as delivery vehicles in combined photothermal therapy [11] and for efficient drug delivery to cancer cells [12].

A significant amount of research has been conducted on the synthesis of AuNPs using polyamidoamine (PAMAM) dendrimers as stabilizers [13,14,15]. These dendrimers are excellent candidates for preparing metal nanoparticles due to their well-defined structure and robust stabilization properties. AuNPs stabilized with PAMAM dendrimers show significant promise in medical applications, particularly for cancer imaging [16] and theranostics [17], where the same platform is used for both therapy and diagnostics. This dual functionality allows for real-time monitoring of treatment efficacy and disease progression [18,19]. These complexes have proven effective as contrast agents in computed tomography (CT) imaging, surpassing the radiation absorption capabilities of traditional iodine-based agents [20]. AuNPs are excellent contrast agents due to their high atomic number, which enhances image contrast. When combined with PAMAM dendrimers, they can provide better signal intensity and help in the early detection of diseases like cancer [18,21].

Additionally, AuNPs/PAMAM complexes can act as carriers for drugs or genes. PAMAM dendrimers can encapsulate drugs efficiently due to their branched structure, and when combined with AuNPs, they can further improve drug loading and controlled release. The attachment of polymers or specific antibodies to these stabilized nanoparticles enhances their affinity for tumor cell receptors, promoting endocytosis within tumor tissues. This innovative approach offers new possibilities for targeted drug delivery and precise imaging in cancer treatment.

Maintaining the stability of AuNPs within the dendrimer matrix is crucial. There is a risk of aggregation, which can affect the delivery and imaging capabilities. The formation of loose PAMAM multilayers and their desorption has been observed, indicating potential stability issues [22]. The stability of these nanoparticles can be influenced by environmental factors such as pH and ionic strength, which can lead to their premature release or degradation [22,23].

The aim of this work is to develop a rapid, two-step synthesis of gold nanoparticles stabilized with PAMAM dendrimers of the fourth generation (G4), which are stable for a long period after synthesis, up to 18 months. The synthesis includes two steps: short sonication followed by exposing the solution that has a reducer to microwaves, which results in the formation of stable nanoparticle colloids. PAMAM-stabilized gold nanoparticles were fully characterized just after synthesis, as well as after 18 months. The methods used to characterize nanoparticles include ultraviolet-visible spectroscopy (UV-Vis), Fourier Transform Infrared Spectroscopy (FTIR), dynamic light scattering (DLS), atomic force microscopy (AFM), and transmission electron microscopy (TEM). Additionally, the cytotoxicity of the produced colloids was assessed immediately after synthesis and after 18 months in comparison with commercially available sodium citrate-stabilized gold nanoparticles, which were tested on the breast cancer MCF-7 cell line using the tetrazolium salt reduction test (XTT).

## 2. Results

### 2.1. Characterization of AuNPs/PAMAM

**Figure 1:**
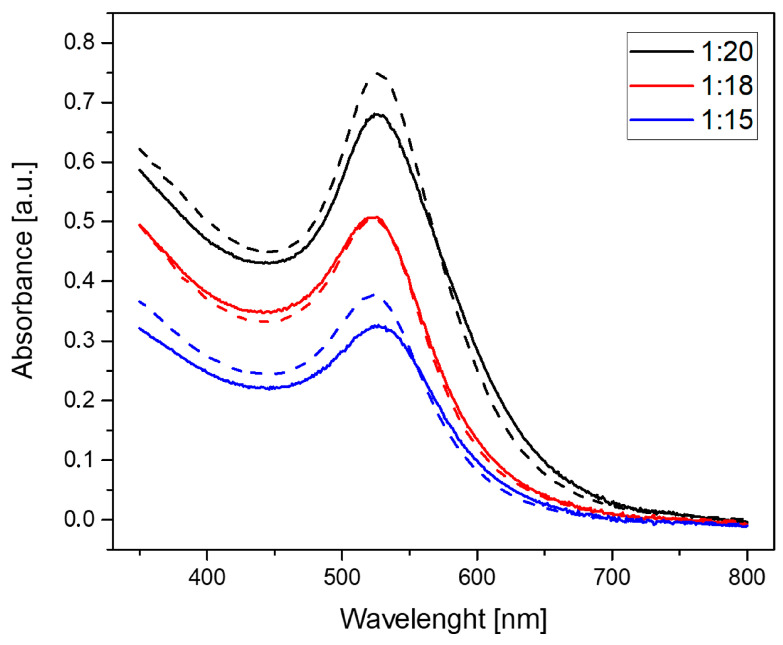
UV-Vis spectra of synthesized AuNPs/PAMAM at varying PAMAM G4:HAuCl_4_ concentration ratios. Solid curves represent spectra collected immediately after synthesis, while dashed curves show spectra collected 18 months post-synthesis.

Figure 1 shows the UV-Vis spectra with the absorbance peak at about 525 nm for each sample post-synthesis and after 18 months. The measurements after 18 months revealed an increase in absorbance intensity for 1:15 and 1:20 samples. The absorbance intensity did not change for the 1:18 sample.

**Figure 2:**
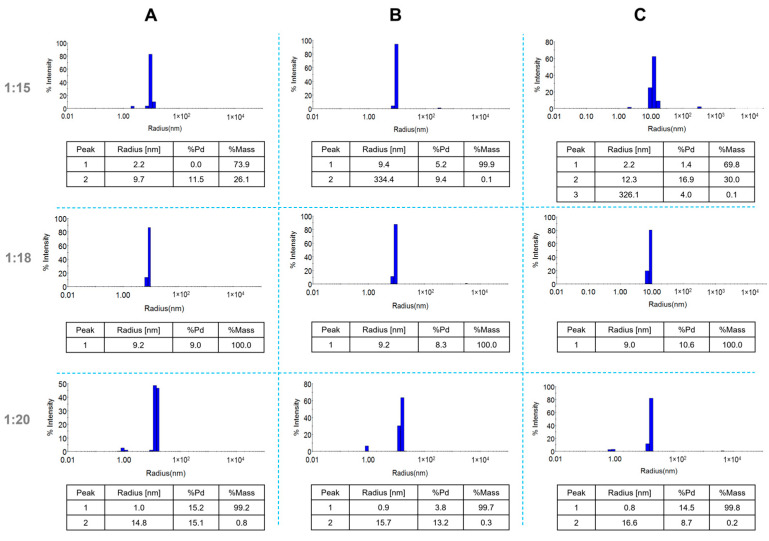
DLS size distribution of AuNPs/PAMAM at varying PAMAM G4:HAuCl_4_ concentration ratios immediately after synthesis (**A**), one month post-synthesis (**B**), and 18 months post-synthesis (**C**).

Figure 2 illustrates the apparent hydrodynamic distribution of synthesized nanoparticles in suspensions with ratios of 1:15, 1:18, and 1:20 at three different time points: immediately after synthesis (Figure 2A), one month post-synthesis (Figure 2B), and 18 months post-synthesis (Figure 2C).

For the 1:15 and 1:18 samples, the primary population of nanostructures exhibited an apparent hydrodynamic radius of approximately 9 nm. Additionally, the 1:15 sample displayed a secondary peak with an apparent radius of 2.2 nm. The 1:20 sample revealed two populations with hydrodynamic radii of approximately 1 nm and 15 nm. All samples demonstrated low polydispersity values, ranging from 5.2% to 16.9%. Zeta potential measurements yielded values of 43.0 (±2.9) mV, 35.9 (±3.4) mV, and 30.3 (±0.9) mV for the 1:15, 1:18, and 1:20 samples, respectively. Notably, after 18 months, all synthesized colloids exhibited reduced zeta potential values (Table 1).

**Figure 3:**
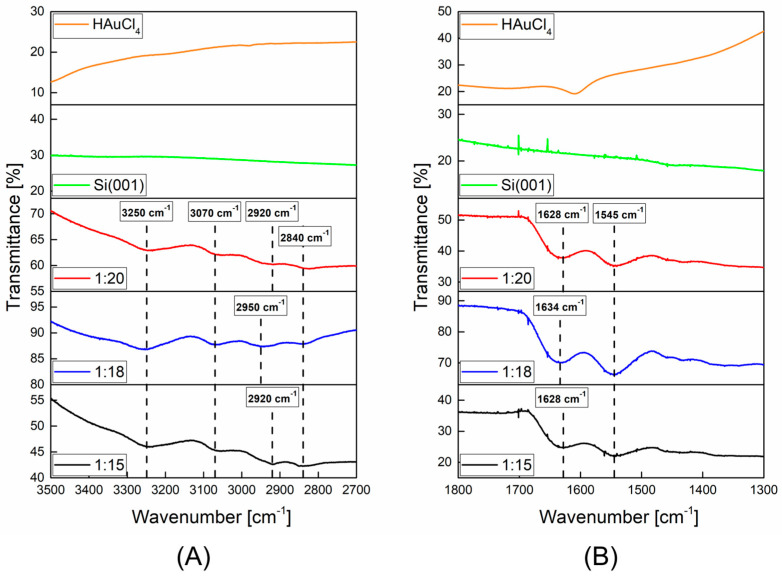
FTIR spectra of AuNPs/PAMAM with different PAMAM G4:HAuCl_4_ concentration ratios along with the Si(001) substrate and HAuCl_4_ of analyzed ranges: (**A**) 3500–2700 cm^−1^ and (**B**) 1800–1300 cm^−1^.

For the chemical characterization of AuNPs/PAMAM, the infrared spectroscopy was performed (Figure 3). The FTIR spectra demonstrated broad vibration peaks at the maximum at 3250 cm^−1^, 3070 cm^−1^, 2920 (2950 for 1:18 sample) cm^−1^, 1628 (1634 for 1:18 sample) cm^−1,^ and 1545 cm^−1^.

**Figure 4:**
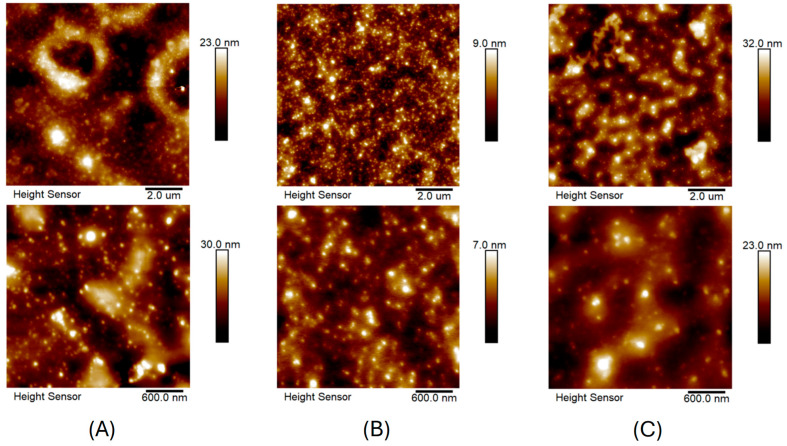
Surface topography images of AuNPs/PAMAM of 1:15 (**A**), 1:18 (**B**), and 1:20 (**C**) samples deposited on mica substrate. The upper and lower (**A**–**C**) images correspond to the 10 × 10 and 3 × 3 μm scan sizes, respectively.

Figure 4 shows the AFM images of AuNPs/PAMAM samples deposited on a mica substrate. The upper images (Figure 4A–C) show the surface topography for a scan size of 10 × 10 μm. For a better visualization of AuNPs/PAMAM, images with a scan size of 3 × 3 μm (bottom images) are also included. From AFM images, numerous nanostructures with sizes below 100 nm can be seen. During the sample drying of AuNPs/PAMAM on mica, the complexes changed and probably flattened (increasing the size in X and Y directions) or aggregated. From the AFM images, it can be concluded that the most separated nanoparticles and the most homogeneous surface over the entire scanning area were obtained for the 1:18 sample.

**Figure 5:**
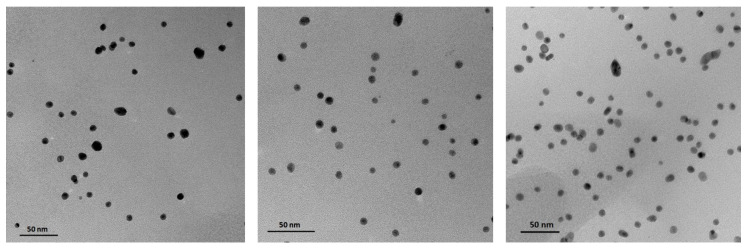
TEM images of AuNPs/PAMAM of the 1:18 sample taken from 3 randomly selected areas.

For TEM investigation (Figure 5), the 1:18 sample was selected. The images show spherical and well-dispersed nanoparticles with a diameter of about 10 nm.

### 2.2. Investigation of AuNPs/PAMAM Cytotoxicity on MCF-7 Cell Line

**Figure 6:**
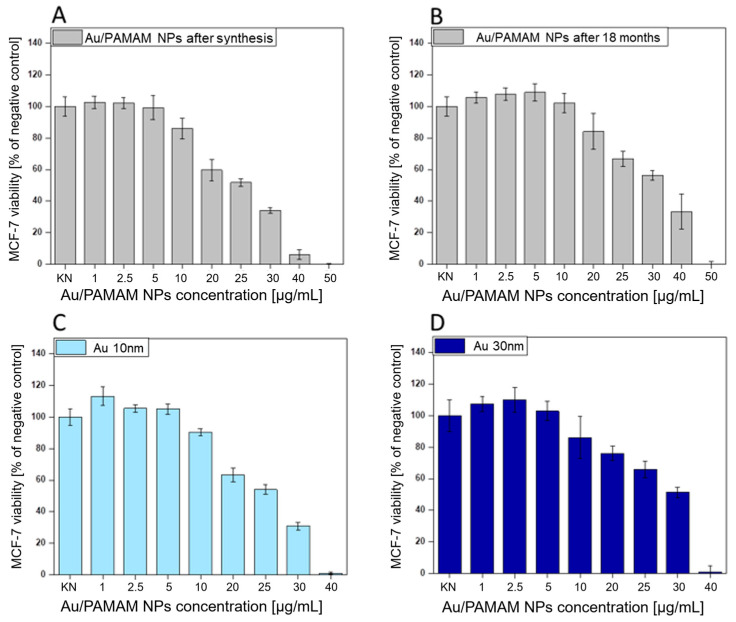
The viability of MCF-7 cells measured by XTT after 24 h incubation with 1:18 AuNPs/PAMAM G4 sample used at selected concentrations just after synthesis (**A**), 18 months after synthesis (**B**), and commercially available AuNPs with diameters of 10 nm (**C**) and 30 nm (**D**), respectively. Data are presented as a mean ± standard error of the mean.

The viability of MCF-7 cells exposed to AuNPs/PAMAM G4 of the 1:18 sample immediately after synthesis and 18 months after synthesis, as well as commercially available gold nanoparticles of 10 nm and 30 nm size, is presented in Figure 6.

Au/PAMAM complexes immediately after synthesis are more toxic (noticeable above the concentration of 10 µg/mL). From the obtained viability graphs, EC_10_, EC_25,_ and EC_50_ concentrations were calculated, which are gathered in Table 2. EC_10_, EC_25_, and EC_50_ correspond to the 90, 75, and 50% of the control cellular viability.

## 3. Discussion

In order to verify the presence of AuNPs/PAMAM and their stability, UV-Vis and DLS techniques were used. UV-Vis spectra were collected right after synthesis and after 18 months of storage at room temperature (Figure 1). The specific band of UV-Vis spectra for the surface plasmon resonance (SPR) of gold nanoparticles occurred in a broad range of 500–550 nm [13]. The narrow absorbance peak at 525 nm suggested the presence of spherical AuNPs of 4–50 nm diameter [24]. The absorbance intensity of the 1:18 sample remained unchanged, indicating high stability over 18 months of storage. In contrast, at ratios of 1:15 and 1:20, the intensity of the SPR peak increased after 18 months, suggesting an increase in the concentration of nanoparticles in the solutions over time [25].

The zeta potential values for colloids immediately after synthesis are quite high, ranging from 30.3 to 43.0 mV (Table 1). These results align with the literature reports [26], which indicate a zeta potential of approximately 41 (±3) mV for AuNPs stabilized with PAMAM G4 dendrimers. Interestingly, after 18 months, the zeta potential values for the same colloids decrease to 13.7, 15.7, and 18.6 mV, respectively, suggesting electrokinetic changes in the nanoparticles that may affect their biological activity. DLS analysis (Figure 2) showed that the apparent hydrodynamic radius of the gold nanoparticles in the 1:18 sample remained around 9 nm even after 18 months. For the 1:15 sample, a primary peak at 9.7 nm and a secondary peak at 2.2 nm were observed, indicating the presence of additional products such as stabilizers and synthesis by-products. Similarly, the 1:20 sample exhibited two hydrodynamic radius populations: one at approximately 1 nm, likely representing residual synthesis products, and another at 15 nm, corresponding to the nanoparticles. All synthesized colloids remained stable for up to 18 months, as confirmed by DLS measurements. AFM imaging revealed spherical nanoparticles or their agglomerates below 100 nm. However, AFM has limitations, as imaging is performed after drying the sample, causing Au/PAMAM conjugates to agglomerate and flatten, increasing their size in the X and Y axes while decreasing in the Z axis [27].

DLS results indicate that the 1:18 sample remains stable for up to 18 months, prompting TEM imaging for this case. TEM images revealed nanoparticles approximately 10 nm in diameter. The size of the gold nanoparticles observed in TEM images corresponds to the metallic core, as low-generation dendrimers are not visible in TEM. The literature shows that for higher generation PAMAM dendrimers, such as G8-G10, metal nanoparticles are located within the PAMAM structures [28]. The discrepancy in nanostructure diameter values obtained from TEM and DLS techniques arises from TEM’s ability to observe only the gold core of the particles, while DLS measures the entire AuNPs/PAMAM complex [29]. The synthesized nanostructures should be regarded as Au/PAMAM complexes, where the size and shape are influenced by both the gold nanoparticle core and the PAMAM dendrimer shell, with sodium citrate acting as a stabilizing agent.

The FTIR spectra (Figure 3) of AuNPs/PAMAM showed the presence of the dendrimers in all synthesized nanoparticles. The band at 3250 cm^−1^ corresponds to the N-H stretching vibration arising from the PAMAM stabilizing layer. The next peak at 3070 cm^−1^ is assigned to the N–H bending vibration. For the 1:18 sample, the bands at 2950 cm^−1^ and 2840 cm^−1^ indicated unsymmetrical and symmetrical stretching vibration of methylene [30]. Moreover, spectroscopy bands analysis revealed a shift in the first-mentioned band to lower wavenumbers (2920 cm^−1^) for 1:15 and 1:20 AuNPs/PAMAM colloids. According to literature data [31], the amide peaks at 1630 cm^−1^ and 1540 cm^−1^ (amide I and amide II, respectively) are characteristic of the dendrimer branches. The obtained FTIR results indicate that the characteristic absorption bands of amide I and II for 1:18 Au nanoparticles colloid are shifted to 1634 cm^−1^ and 1545 cm^−1^, respectively, further confirming the involvement of –NH_2_ of G4 PAMAM in the formation of the conjugates. A similar shift in the amide II absorption band was detected for 1:15 and 1:20 colloids. It confirms that PAMAM G4 is indeed formed on AuNPs.

Another aspect of our work was to investigate the toxicity of AuNPs/PAMAM on the human breast adenocarcinoma cell line. The cytotoxicity of Au/PAMAM G4 on human breast cells was dose-dependent (Figure 6). When applied immediately after synthesis at a concentration of 10 µg/mL, Au/PAMAM reduced cell viability to 90% compared to control (untreated) cells. After 18 months, the AuNPs/PAMAM exhibited reduced toxicity, with 90% cell viability achieved at a concentration of 15 µg/mL.

Direct contact of AuNPs/PAMAM with cells in the medium, as well as their penetration into the cells, can initiate various processes leading to pathological changes and cell death. Metallic nanoparticles can accumulate on or just below the cell membrane [32,33,34,35]. The toxicity of the synthesized nanoparticles is significantly influenced by PAMAM dendrimers, with their cytotoxicity largely depending on the number and charge of surface functional groups. PAMAM dendrimers terminated with amines have their surface groups (primary amines) protonated under physiological conditions. Cationic dendrimers are typically highly toxic, whereas anionic and uncharged dendrimers exhibit little to no toxicity. The toxicity of PAMAM dendrimers is widely discussed across various cell lines. The cytotoxicity of cationic PAMAM dendrimers is believed to result from interactions between the positively charged dendrimers and the negatively charged cell surfaces [36,37]. From the work [38], it was shown that dendrimer-lipid conjugates with a higher number of attached chains were less effective at reducing the cytotoxicity of the cationic dendrimers. Similarly, conjugation of G4 PAMAM dendrimers with 4 PEG chains resulted in a marked reduction in cytotoxicity.

The toxicity of AuNPs/PAMAM G4 complexes should be primarily considered in terms of dendrimer functional groups. Based on our results, after 18 months, AuNPs/PAMAM are less toxic (higher EC_10_, EC_25_, EC_50_ values), likely due to the reduced activity of polycationic groups in the dendrimers. Additionally, the decreased zeta potential values after 18 months (Table 1) suggest temporal changes in the functional groups of PAMAM dendrimers. Continuing, it suggests a hypothesis related to surface modification of the synthesized colloids, which may result from the neutralization of residual cationic dendrimer groups by residual citrate ions. The reduction in positive charge of dendrimers (visible as a decrease in zeta potential) results in decreased toxicity of the studied AuNPs/PAMAM complexes. Furthermore, the cytotoxicity of the synthesized complexes was compared to that of commercially available nanoparticles stabilized with sodium citrate, with diameters of 10 and 30 nm. Surprisingly, AuNPs/PAMAM immediately after synthesis are as toxic as commercially available nanoparticles stabilized with sodium citrate with a diameter of 10 nm (Table 2). Notably, AuNPs/PAMAM conjugates synthesized using a different model but of similar size are much more toxic to human umbilical vein endothelial cells (HUVEC) [29] than the tested conjugates on the breast adenocarcinoma MCF-7 cell line. This suggests that cancer cell lines are more resistant to the effects of nanostructures and that differences in the synthesis process of these conjugates may influence their chemical activity and toxicity.

## 4. Materials and Methods

Dendrimers used in this study were purchased from Sigma Aldrich (Saint Louis, MO, USA) as a methanol suspension. Polyamidoamine dendrimers of 4th generation (cat. no. 412449) consist of an ethylenediamine core (2-carbon core) with 64 functional primary amino groups on the surface, respectively. These data refer to the specification sheets provided by the manufacturer. According to the manufacturer’s certificates, the high purity of PAMAM solutions was confirmed by infrared spectrum and 13C NMR identity.

Au NPs stabilized with sodium citrate (cat. no. J67188 and J67284), defined as reference, were provided from Sigma Aldrich (Saint Louis, MO, USA). According to the manufacturer’s protocol, these gold nanoparticles are non-functionalized, stable in high salt (30 mM NaCl) for at least 30 min.

### 4.1. Two—Stage Synthesis of AuNPs/PAMAM

Gold (III) chloride hydrate (HAuCl_4_·3H_2_O) from Sigma Aldrich (USA) served as the precursor for synthesizing gold nanoparticles. Fourth-generation PAMAM dendrimers with an ethylenediamine core and 64 primary amino groups, provided in a 10% methanol solution, were employed as stabilizers. Sodium citrate (Chempur, Piekary Śląskie, Poland) was utilized as the reducing agent.

The synthesis of gold nanoparticles followed a modified protocol based on Avila-Salas et al. [39]. Initially, the PAMAM G4 solution was dried at 60 °C for 30 min to eliminate methanol. Subsequently, 0.25 mL of HAuCl_4_ solutions at concentrations of 1.5 mM, 1.8 mM, and 2.0 mM were each combined with 0.25 mL of a 0.1 mM aqueous PAMAM G4 solution and sonicated for 3 min at room temperature. In the next step, 0.25 mL of sodium citrate was added to a third of the gold molar concentration mixture of chloroauric acid to reduce the Au complex. The samples were then subjected to microwave irradiation for three cycles of 10 s each. The power of the microwaves was 800 W, while the power used for the sonication of chloroauric acid/PAMAM was 180 W. The appearance of a red colloid confirmed the formation of gold nanoparticles. This two-step synthesis method, incorporating sonication and microwave irradiation, significantly reduces synthesis time and produces monodisperse gold nanoparticles that remain stable for up to 18 months, as compared to our previous work [29].

### 4.2. Ultraviolet-Visible Spectroscopy

The optical properties of AuNPs/PAMAM complexes were analyzed using UV-Vis spectroscopy with an Ultrospec 2100 Pro spectrophotometer (GE, Farnborough, UK). The colloids were placed in disposable cuvettes and measured at room temperature immediately following synthesis and again 18 months later.

### 4.3. Dynamic Light Scattering Technique and Zeta Potential Measurements

The particle size distribution of the AuNPs/PAMAM complexes was determined using dynamic light scattering with a Dyna Pro Nano Star device (Wyatt, Santa Barbara, CA, USA). Samples were placed in disposable cuvettes immediately after synthesis, and measurements were conducted in five replicates, each consisting of 10 acquisitions. The DLS measurements were performed under the following conditions: temperature of 25 °C, laser wavelength of 663 nm, laser power of 100 mW, and scattering angle of 90°. The apparent hydrodynamic radius for all populations was calculated based on the obtained histograms and regularization analysis [40].

Zeta potential measurements were carried out using a Zetasizer Nano device (Malvern, UK). These measurements were performed in triplicate, with each triplicate consisting of 20 acquisitions. AuNPs/PAMAM colloids were placed in disposable folded capillary zeta potential cells (Cat number DTS1070, Malvern, UK) and measured both immediately after synthesis and 18 months later. The measurement parameters included a scattering angle of 173°, wavelength of 663 nm, laser power of 4 mW, and temperature of 25 °C. The study utilized automatic attenuation and voltage selection. Zeta potential values were calculated for the Au material using a refractive index of 0.2 and absorption of 3.32, according to the Smoluchowski model.

### 4.4. Infra-Red Spectroscopy

Fourier Transform Infrared Spectroscopy measurements were conducted using a Nicolet iS50 FT-IR spectrometer (Thermo Scientific, Waltham, MA, USA) equipped with an attenuated total reflectance (ATR) device, operating at a resolution of 1 cm^−1^. The colloidal samples were deposited on cleaved Si(001) wafers and allowed to dry at room temperature.

### 4.5. Atomic Force Microscopy

The visualization of AuNPs/PAMAM complexes was carried out using a BioScope Resolve atomic force microscope (Bruker, Billerica, MA, USA) in Peak Force Tapping mode with a Scan Asyst Probe (Bruker, USA). The samples were prepared by depositing AuNPs/PAMAM on mica, following the procedure outlined in [29]. The imaging parameters included scan sizes of 10 × 10 μm and 3 × 3 μm, with scan rates of 0.5 Hz and 1 Hz, respectively.

### 4.6. Transmission Electron Microscopy

Transmission Electron Microscopy analysis was conducted using a Talos F200X microscope (FEI, Eindhoven, The Netherlands) operating at an acceleration voltage of 200 kV. The selected colloid sample (designated as 1:18) was deposited onto carbon-coated copper grids (300 mesh) and left to dry at room temperature.

### 4.7. Cell Culture and Cytotoxicity Measurements (XTT Assay)

The human breast adenocarcinoma MCF-7 cell line (HTB-22™) was sourced from ATCC (Manassas, VA, USA), along with Dulbecco’s Modified Eagle Medium (DMEM), Fetal Bovine Serum (FBS), a Penicillin/Streptomycin cocktail, and a 0.25% (*w*/*v*) Trypsin—0.53 mM EDTA solution. Phosphate-buffered saline (PBS) was obtained from Gibco (Norristown, PA, USA), and the tetrazolium salt XTT (2,3-bis[2-methoxy-4-nitro-5-sulfophenyl]-2H-tetrazolium-5-carboxanilide) was purchased from Biological Industries (USA). MCF-7 breast cancer cells were routinely cultured in complete DMEM supplemented with 10% FBS. All cell culture reagents were handled under sterile conditions. The cells were maintained in 75 cm^2^ flasks within a humidified incubator set at 37 °C with a 5% CO_2_ atmosphere. Subculturing was performed every 3–4 days, coinciding with the appearance of the first floating cells in the medium and achieving approximately 80–90% confluence.

The cytotoxicity of AuNPs/PAMAM G4 was evaluated using tetrazolium salt reduction assays, following the supplier’s protocol (Biological Industries, Cromwell, CT, USA). MCF-7 cells were seeded in 96-well plates at a density of 10,000 cells per well. After 24 h of incubation, the culture medium was replaced with suspensions of gold nanoparticles at final concentrations of 1, 2.5, 5, 10, 20, 25, 30, 40, and 50 μg/mL in serum-free medium. Parallel experiments were conducted using commercially available reference AuNPs with diameters of 10 and 30 nm. Control cells received serum-free medium without nanoparticles. Following an additional 24 h incubation, cell viability was assessed using the XTT assay. Absorbance at 450 nm was measured for each well using a Victor X4 microplate reader (Perkin Elmer, Waltham, MA, USA). Cell viability was expressed as a percentage relative to control cells. The effective concentrations reducing cell viability to 90%, 75%, and 50% of control levels were calculated and designated as EC_10_, EC_25_, and EC_50_, respectively.

## 5. Conclusions

The presented method of synthesis allows for the preparation of spherical and well-separated, long size-stable AuNPs/PAMAM in an aqueous solution. The synthesis of nanoparticles is based on two steps: the first is a short sonication, and then the use of microwaves, which significantly shortens production time. The developed procedure is repeatable and enables the production of nanoparticles on a large scale. Additionally, XTT assays were conducted to assess the impact of AuNPs/PAMAM on MCF-7 cells, revealing similar toxicity levels to commercially available nanoparticles stabilized with sodium citrate only.

In the future, the developed rapid synthesis may play a crucial role in medical and pharmaceutical sciences, particularly for the application of AuNPs/PAMAM complexes in diagnostics, imaging, and drug delivery systems.

Overall, this research could pave the way for advancements in nanomedicine, leading to new diagnostic tools, imaging techniques, and targeted drug delivery systems that could significantly improve patient outcomes.

## Figures and Tables

**Table 1:** Zeta potential values for colloids as synthesized and 18 months post-synthesis.

Samples	Zeta Potential As-Synthesized [mV]	Zeta Potential 18 Months Post-Synthesis [mV]
1:15	43.0 (±2.9)	13.7 (±0.7)
1:18	35.9 (±3.4)	15.7 (±0.7)
1:20	30.3 (±0.9)	18.6 (±0.6)

**Table 2:** Comparison of AuNPs concentrations. EC_10_, EC_25_, and EC_50_ correspond to the 90, 75, and 50% of the control cellular viability.

Designation	AuNPs/PAMAM After Synthesis [µg/mL]	AuNPs/PAMAM 18 Months After Synthesis [µg/mL]	AuNPs 10 nm [µg/mL]	AuNPs 30 nm [µg/mL]
EC_10_	10 ± 2.3	15 ± 1.5	10.5 ± 1.9	12 ± 2.8
EC_25_	15 ± 2.3	22 ± 1.7	15.5 ± 2.2	19 ± 3.4
EC_50_	24.5 ± 2.8	33 ± 2.0	24 ± 2.7	31.5 ± 4.4

## Data Availability

The original contributions presented in this study are included in the article. Further inquiries can be directed to the corresponding author(s).
